# Clinical characteristics and associated factors of trigeminal neuralgia: experience from Addis Ababa, Ethiopia

**DOI:** 10.1186/s12903-020-01227-y

**Published:** 2020-09-03

**Authors:** Biniyam Alemayehu Ayele, Abenet Tafesse Mengesha, Yared Zenebe Zewde

**Affiliations:** Department of Neurology, School of Medicine College of Health Sciences, Addis Ababa University, PO Box 6396, Liberia Street, Lideta sub-city, Addis Ababa, Ethiopia

**Keywords:** Trigeminal neuralgia, Classical TN, Symptomatic TN, Tooth extraction, Carbamazepine

## Abstract

**Background:**

Trigeminal neuralgia (TN) is considered one of the most painful illnesses known to medical practice. Little is known about TN in Ethiopia. Our study aimed to assess clinical characteristics, treatment, and associated factors of TN.

**Method:**

A cross-sectional study was conducted on a total of 61 patients with confirmed Trigeminal neuralgia visiting outpatient neurology clinics of two government teaching Hospitals and two private health facilities in Addis Ababa, Ethiopia between June 2019 and March 2020.

**Results:**

Our participants’ age range between 21 and 78 years with mean ± SD age of 50.7 ± 14.2 years. Males accounted for 50.8%. Twenty-five (41%) reported a prior history of one or more tooth extraction on the painful side. In the majority (68.9%) of the patient’s right side of the face was affected. The mandibular nerve was the commonly involved branch (47.5%). Fifty-five (90.2%) of patients fulfilled criteria for classical TN and 9.8% had symptomatic TN. The majority of the participants reported mixed types of pain such as burning, lancinating, and electric shock-like. Well defined trigger zone was identified in one-third (36%) of cases. Carbamazepine was the most commonly prescribed drug with a median dose of 600 mg (IQR: 400 – 1000 mg). Two-third of the patients reported prominent satisfaction. The mean (± SD) dose of carbamazepine used to control the pain was significantly higher among those with dental extraction history as compared to those with no history of dental extraction (736 ± 478.6 mg Vs 661.1 ± 360.4 mg, respectively, T = − 2.06, *p* = 0.04 95% CI:-213.41 to − 2.98). A statistically significant number of patients who had single branch involvement reported prominent satisfaction with their treatment as compared to those who had more than one branch involvement. (95% CI: 1.3–3.8: *p* = 0.006).

**Conclusion:**

The majority of our patients had Classical TN in the mandibular nerve distribution on the right side of the face and well satisfied with carbamazepine only treatment. Furthermore, we observed a higher proportion of dental extraction among our patients, hinting at the scale of miss and delayed-diagnoses. Thus, we recommend conducting a well-designed prospective study to support our findings.

## Background

Trigeminal neuralgia (TN) is a relatively rare neuropathic disorder, characterized by extremely painful episodic facial pain involving one or more trigeminal nerve branches. Based on the International Classification of Headache Disorders- 3rd Edition (ICHD-3) TN is classified into three: Classical TN, Secondary TN, and Idiopathic TN, based on the presence or absence of an apparent disease process that could explain the neuralgia [1]. Considering the wide variation in its clinical symptomatology, delayed or misdiagnosis is common. It is not uncommon for patients with Trigeminal neuralgia to be managed by non-neurologist health professionals such as dentists, general practitioners, internists, anesthesiologists, and neurosurgeons [2].

Quality of pain related to TN is described as electric shock-like, sharp, stabbing, or shooting, often triggered by immaterial sensory input such as washing face, brushing, wind blow, and talking [3]. The exact pathophysiology of TN is still largely uncharted. However, current consensus regarding the underlying etiology of TN orbits around focal demyelination of trigeminal nerve root entry zone as a result of compression by an aberrant loop of artery or vein often referred to as Classical TN (CTN). Furthermore, it has been demonstrated that TN is more common in patients with multiple sclerosis, which is often resulted in Symptomatic or Secondary TN (STN). Few published reports support the possible inheritance pattern of Trigeminal neuralgia, supporting the role of genetics as etiology of TN. Familial classic trigeminal neuralgia (FCTN), may account for 2% of cases of TN [4].

The variabilities related to clinical features of trigeminal neuralgia may be attributable to environmental and genetic influences. Information on the clinical pattern, treatment responses, and associated factors of TN are rare in the African continent, especially in Sub-Saharan Africa (SSA) countries [5]. To the author’s knowledge, so far there is no published data from Ethiopia regarding TN. Therefore, the objective of this study was to determine the clinical characteristics, treatment, and associated factors of Trigeminal neuralgia patients attending tertiary level government Hospitals and private health facilities in Addis Ababa, Ethiopia. Likewise, finding from this observation will contribute to the existing understanding regarding Trigeminal neuralgia, as data from Sub-Saharan Africa was lacking regarding TN.

## Methods

### Study objective and study setting

Our study aimed to assess clinical characteristics, treatment, and associated factors of TN. The study was conducted at the outpatient neurology clinics of two government teaching hospitals (Tikur Anbessa Specialized Hospital (TASH) and Zewditu Memorial Hospital (ZMH) and two private health facilities (Bethezata General Hospital and Yehuleshet Specialty Clinic) in Addis Ababa, Ethiopia.

### Study period and design

A cross-sectional, observational study was conducted between June 2019 and March 2020. All 61 patients who were age > 18 years, having a clinical diagnosis of TN as per the International Classification of Headache Disorders-3rd Edition (ICHD-3) criteria [1] and consented to participate were included. Treatment satisfaction was assessed in three categories, no satisfaction, mild satisfaction, prominent (moderate + significant) satisfaction [6, 7] (Table 1).

**Table 1:** Trigeminal neuralgia diagnostic criteria based on ICHD-3

**A**. Recurrent paroxysms of unilateral facial pain in the distribution(s) of one or more divisions of the trigeminal nerve, with no radiation beyond, and fulfilling criteria **B** and **C**.	
**B**. Pain has all of the following characteristics:	
1. Lasting from a fraction of a second to two minutes	
2. Severe intensity	
3. Electric shock-like, shooting, stabbing or sharp in quality	
**C**. Precipitated by innocuous stimuli within the affected trigeminal distribution	
**D**. Not better accounted for by another ICHD-3 diagnosis	

ICHD-3: International Classification of Headache Disorders-3rd Edition criteria

### Data collection tool and procedure

A semi-structured questionnaire (Supplementary file 1) assessing the socio-demographic, clinical characteristics, treatment type and dose, and treatment satisfaction level were administered to each participant by three board certified neurologists. Patients Electronic Medical Record (EMR) was also reviewed for additional clinical data.

### Data analysis

Completed questionnaires were cleaned and entered into Statistical Package for Social Sciences (SPSS) Version 25 for analysis. We used descriptive statistics with frequency and proportion for categorical data and mean, median, interquartile range (IQR), range, and standard deviation (SD) for continuous variables. Associations between selected variables were tested using Student’s t-test and Fisher’s exact or Chi-square tests. Results were interpreted and presented using appropriate tables and figures. Variables with *p* values < 0.05 were considered statistically significant.

## Results

### Demographics, duration of illness, dental extraction, anatomy, and sensory abnormalities

A total of 61 patients with Trigeminal neuralgia who visited four health facilities over nine months were included in this study. The mean ± SD age of the study participants was 50.7 ± 14.2 years (Range: 21–78 years). Males accounted for 50.8%. The mean ± SD of for disease duration was 5.7 ± 3.9 years.

Twenty-five (41%) of the patients reported removal or filling of one or more lower molar/ premolar tooth. In two-third (68.9%) of the patient’s right side of the face was affected, 29.5% left side was affected, at the same time, the bilateral face was involved in 1.6%. In half of the patients, the mandibular branch (V3) was involved, followed by the involvement of both maxillary and mandibular branch (V2 + V3) in 34.4% of the patients. No patient demonstrated the involvement of an isolated ophthalmic branch (V1). Half of the patients had a diagnosis of TN for < 5 years, while 11.5% of them were living with TN for more than 10 years. One patient (1.6%) had ipsilateral facial sensory abnormality (Table 2).

**Table 2:** Demographics, duration of illness, tooth extraction, anatomy, and sensory abnormality

Characteristics	Frequency (N)	Percent (%)
Age category		
21–40 years	18	29.5
41–60 years	26	42.6
> 60 years	17	27.9
Gender		
Male	31	50.8
Female	30	49.2
Duration of illness		
< 5 years	31	50.8
5–10 years	23	37.7
> 10 years	7	11.5
Dental extraction		
Yes	25	41
No	36	59
Side of the face affected		
Right sided	42	68.9
Left sided	18	29.5
Bilateral	1	1.6
Trigeminal nerve branches		
V2	6	9.8
V3	29	47.5
V2 + V3	21	34.4
V1 + V2 + V3	5	8.2

V_1_: Ophthalmic branch, V_2_: Maxillary branch, and V_3_: Mandibular branch

### Classification, pain characteristics, autonomic symptoms, and pain during sleep

Fifty-five (90.2%) of our study participants fulfilled the criteria of Classical/Idiopathic TN (CTN/ITN), while 9.8% had Symptomatic TN (STN) secondary to CPA meningioma, brainstem cavernoma, and Multiple sclerosis. The average age of patients with CTN/ ITN (51.5 ± 14 years) was higher compared to patients with STN (45.3 ± 14.7 years). Out of six patients with diagnoses of STN, 83.3% were age below 60 years. However, no statistically significant agreement was observed between the age category and classification of TN (Table 3). Most of the participants (26.2%) reported mixed quality of pain such as burning, lancinating, and electric shock. Twenty-two (36%) of them reported having a trigger zone. Among these, the ipsilateral angle of mouth accounted for 21.3%, nasolabial fold accounted for 8.2%, while lower mandibular edge and lower gum accounted for 4.9 and 1.6%, respectively. The majority of the patients reported mixed symptoms such as cold wind blowing, chewing, drinking cold or hot drinks, and having or touching triggering the pain (Table 4). Three (4.9%) reported the pain was associated with autonomic symptoms, while 16.4% reported their pain occasionally wakes them up from sleep. Among the total of 61 study participants, fifteen (24.6%) reported their pain to worsen during cold and rainy seasons.

**Table 3:** Association between classification of Trigeminal neuralgia and different age category

	Age Category			P value
	20–40 years	41–60 years	> 60 years	
Classification of TN	Frequency (%)	Frequency (%)	Frequency (%)	0.5
Classical TN	15 (24.6)	24 (39.3)	16 (26.2)	
Symptomatic TN	3 (4.9)	2 (3.3)	1 (1.6)	
Total	18 (29.5)	26 (42.6)	17 (27.9%)	

*TN* Trigeminal neuralgia

**Table 4:** Classification, pain characteristics, autonomic symptoms, and pain during sleep

Characteristics	Frequency (Yes)	Percent (%)
Quality of pain		
Feeling of being injected with red hot needle	13	21.3
Burning	10	16.4
Lancinating pain	15	24.6
Electric shock like	7	11.5
Mixed features	16	26.2
Total	61	100
Triggering zone		
Ipsilateral angle of mouth	13	21.3
Ipsilateral nasolabial fold	5	8.2
Ipsilateral lower mandibular edge	3	4.9
Ipsilateral lower gum	1	1.6
Total	22	36
Triggering factors		
Mixed triggering factors	37	60.6
Cold wind blowing	4	6.6
Chewing	5	8.2
Drinking cold or hot drinks	3	4.9
Shaving or touching	4	6.6
Non-specific	8	13.1
Total	61	100
Classification of Trigeminal neuralgia		
Classical TN	55	90.2
Secondary TN	6	9.8
Total	61	100

*TN* Trigeminal neuralgia

### Treatments, treatment satisfaction, and family history

**Fig. 1:**
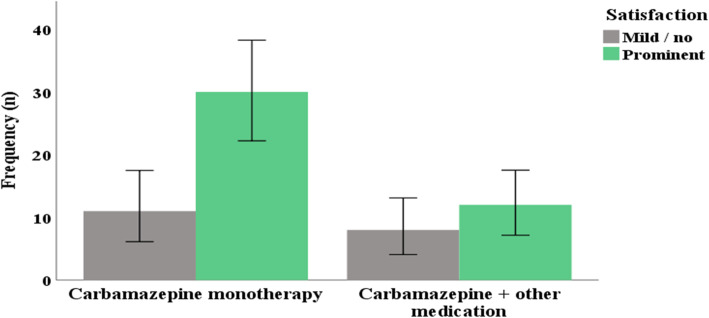
Bar graph showing association between treatment satisfaction and carbamazepine monotherapy or carbamazepine combination with other drugs

Carbamazepine was the commonest prescribed drug among the participants. Forty-one (67.2%) of the patients were on Carbamazepine alone, while 11.5, 9.8, 6.6% were on Carbamazepine + NSAIDs, Carbamazepine + Amitriptyline, and Carbamazepine + Gabapentin, respectively. Few patients were shifted to non-Carbamazepine drugs because of Carbamazepine-induced adverse effects. The median carbamazepine dose of the study participants was 600 mg (IQR: 400 – 1000 mg, range: 0 – 2000 mg). Forty-two (68.8%) of study participants reported having prominent satisfaction with pain medication, while 31.1% reported mild to no satisfaction with their treatment. There is no statistically significant association between treatment regimen and satisfaction (*p* = 0.52). Majority of patients on carbamazepine monotherapy reported prominent satisfaction compared to those patients on combination therapies (Fig. 1). Among a total of 61 patients 1.6% reported a positive family history of TN.

### Association between carbamazepine doses and tooth extraction

**Fig. 2:**
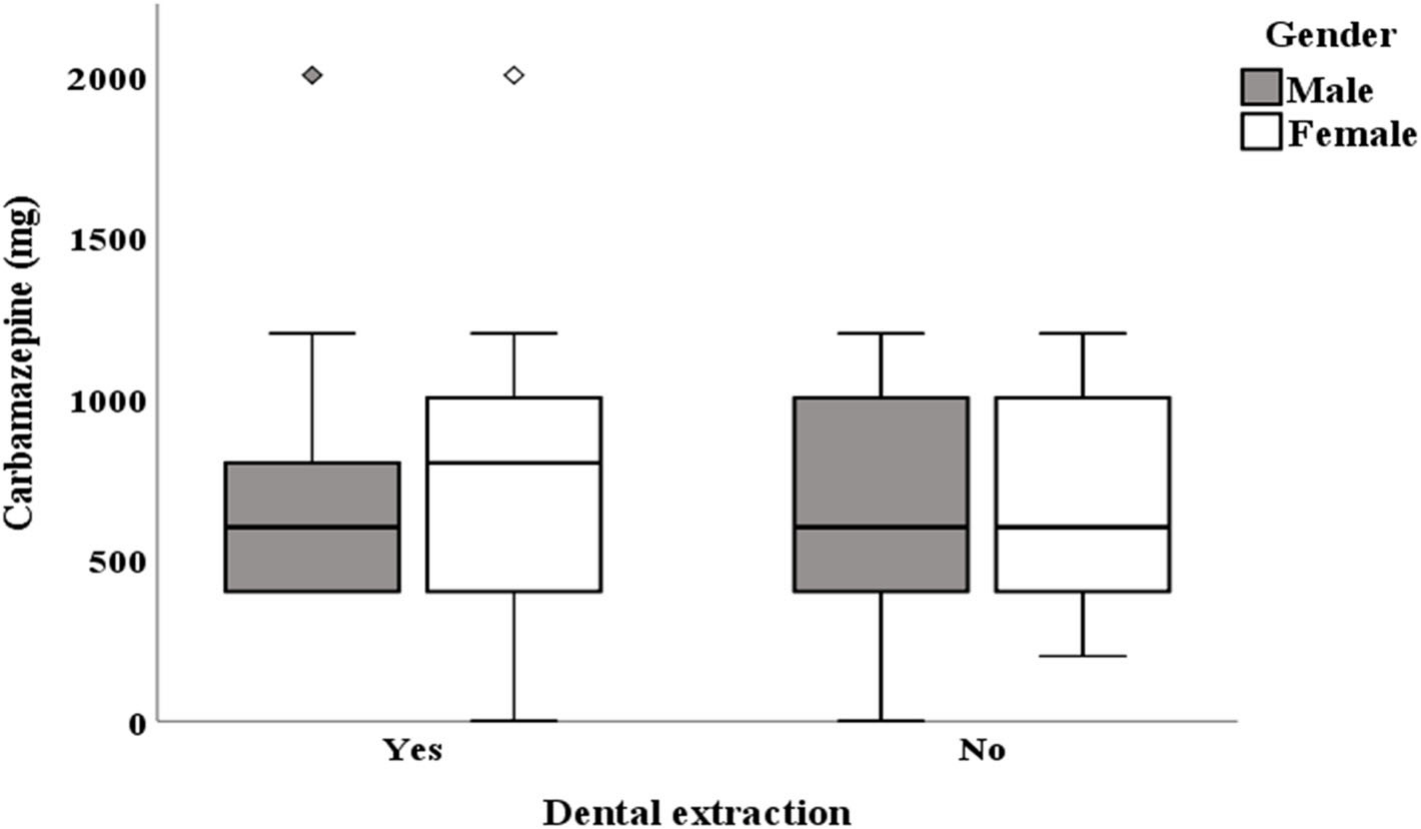
Box and whiskers plot showing gender distribution of mean carbamazepine dose among individuals having history of dental extraction or not

We hypothesized that; among our study participants those patients who had a positive history of tooth extraction will require higher doses of carbamazepine compared to those with no history of tooth extraction to relieve their facial pain. On student t-test analysis, we observed statistically significant association between higher doses of carbamazepine (CBZ = 800 mg) and history of tooth extraction (*t* = − 2.06, *p* = 0.04, 95% CI:-213.41 to − 2.98) (Fig. 1). Females patients who had history of dental extraction tend to be on higher doses of carbamazepine compared to their male counter parts. In contrary, no carbamazepine dose difference was observed among males and females with no history of dental extraction (Fig. 2).

### Association between treatment satisfaction level and gender, branch involved, and tooth extraction

We performed Pearson’s Chi-square test or Fisher exact test to see the association between treatment satisfaction (mild to no satisfaction & prominent satisfaction) and different categorical variables such as gender, branch of the trigeminal nerve (single or multiple branches) and history of tooth extraction. Those patients having a single branch of trigeminal nerve (V2 or V3) involvement reported prominent satisfaction with their treatment as compared to those with multiple branch involvement (*p* = 0.006, 95% CI: 1.3–3.8). Gender and history of tooth extraction did not show statistically significant agreement with treatment satisfaction with *p* = 0.7 and *p* = 0.6, respectively (Table 5).

**Table 5:** Association between treatment satisfaction and gender, branch involved, and tooth extraction

	Treatment satisfaction		X^2^- test [95% CI]	P value
	Mild/No satisfaction	Prominent satisfaction		
	Frequency (%)	Frequency (%)		
Gender				
Male	9 (29)	22 (71)	1	0.7
Female	10 (33.3)	20 (66.7)	1.1 [0.6–1.8]	
Branch Involved				
Single branch	6 (31.6)	29 (69)	2.2 [1.3–3.8]	**0.006**
Multiple branch	13 (68.4)	13 (31)	1	
Dental extraction				
Yes	7 (28)	18 (72)	0.8 [0.4–1.7]	0.6
No	12 (33.3)	24 (66.7)	1	

## Discussion

The mean age of our study participants, the proportion of Classical TN to Symptomatic TN, predominant right side involvement were consistent with regional and global figures [2, 8, 9] Trigeminal neuralgia most often affects females compared to male [2]. However, our study showed slight male predominance (50.8%), this is consistent with the regional report [5, 10]. This difference could be explained with the fact that males tend to seek more medical attention for their illnesses as compared to females in Sub-Saharan countries [5, 9, 10]. Symptomatic TN was commonly seen among younger patients, this is consistent with previous reports [9, 11]. A higher proportion of our patients had a history of dental extraction, a sign of initial visit to the dental physician before the diagnosis of Trigeminal neuralgia was made. This finding is similar to report from Thailand [8] where 40.5% reported a history of tooth extraction. The mandibular nerve was the commonest branch to be affected, followed by a mixed mandibular + maxillary branch, while the ophthalmic branch was not involved in any of our study participants, which is in line with previous reports [12, 13].

The diagnosis of TN is established based on its characteristic clinical symptoms and investigations are only recommended in atypical presentations. While some of our participants primarily characterized their pain as “lancinating”, “feeling of being injected with a red hot needle”, “burning” and “electric shock-like” in descending order, most (26.2%) described it in mixed terms. A similar study from Thailand reported, mixed pain quality in 22.3% of TN patients, although the majority of their patients described it as sharp pain and stabbing/lancinating pain [8] The discrepancies in pain characterization could be explained by patients experience and response to an open or closed question which may lead the patient to one specific answer. All of our participants had some form of pain triggering stimuli and multiple factors were attributed by the majority while no specific factor identified in 13.1%. Clear pain triggering zone was identified in only one-third of the patients. These areas include angles of the mouth, nasolabial fold, lower mandibular edge, and lower gum on the painful side. A study from Thailand also showed among the 188 TN patient’s majority reacted to multiple types of stimulus and the most common trigger was chewing [8]. These findings were consistent with reports from Italy, which showed trigger zones were predominantly found around the perioral and nasal region; the authors also recommended utilizing triggers as an essential diagnostic feature of trigeminal neuralgia [14].

Carbamazepine was the most commonly prescribed drug as initial treatment, while a quarter of the patients required the addition of another drug. Two patients were shifted to another drug because of carbamazepine induced adverse reaction [15] Gabapentin, clonazepam, and amitriptyline were also prescribed to those not tolerating carbamazepine [14, 16]. Patients on carbamazepine reported prominent treatment satisfaction [8]. The mean carbamazepine dose of the study participants was 691.8 mg, which is consistent with similar reports [2, 9, 10]. We found that those patients who had history tooth extraction required a higher dose of carbamazepine compared to those without a history of tooth extraction. These findings are indicative of the potential diagnoses delay and suboptimal treatment of patients with TN experience in Ethiopia. This is parallel to previous reports showing a significant proportion of patients with TN initially visit non-neurologist health professionals [2, 5].

Symptomatic TN was commonly observed among younger patients. Similarly, two-thirds of patients with Classical TN are the age below 60 years. These findings were consistent with regional reports [9, 11]. One (1.6%) of our study participants reported a family history of Trigeminal neuralgia. This finding was supported by a cluster of familial classic trigeminal neuralgia reported from Spain [4]. Individuals with the involvement of a single branch of trigeminal nerve were associated with prominent treatment satisfaction compared to patients with multiple branch involvement. However, no significant agreement was observed between treatment satisfaction and gender and history of tooth extraction, which could be attributable to the small sample size. A small proportion of our patients reported associated autonomic symptoms during episodic pain, which in line with a recently published prospective study on 158 patients with TN [17]. A limitation of our study includes small sample size few patients investigated with MRI and absence of high-resolution brain MRI which can detect contact between vessel and nerve root, no electrophysiology study was performed, and failed to utilize validated treatment satisfaction scale.

## Conclusion

Our findings suggest the majority of our patients had Classical TN and satisfied well with carbamazepine alone treatment. However, a quarter of them required the addition of the second drug. Furthermore, we observed a higher proportion of dental extraction among our patients, hinting at the scale of miss-diagnoses and delayed diagnoses. Thus, we recommend conducting well designed a larger prospective study to support our findings.

## Supplementary information

**Additional file 1.** Study questionnaire, semi-structured questionnaire prepared in order to extract the following data: socio-demographic, clinical characteristics, treatment type and dose, and treatment satisfaction.

## Data Availability

All data sets on which the conclusions of this manuscript rely are available as spread excel sheet documents and available from the corresponding author on reasonable request.

## Abbreviations

ICDH-3: International Classification of Headache Disorders-3rd Edition
STN: Symptomatic/Secondary Trigeminal Neuralgia
NSAIDs: Non-steroid anti-inflammatory drugs
FCTN: Familiar Classic Trigeminal Neuralgia
ITN: Idiopathic Trigeminal Neuralgia
CTN: Classic Trigeminal Neuralgia
CHS: College of Health Science
AAU: Addis Ababa University
TN: Trigeminal neuralgia
