# Supplementary material for: Clinical characteristics and associated factors of trigeminal neuralgia: experience from Addis Ababa, Ethiopia
Source: BMC Oral Health. 2020 Sep 3;20:244. doi: 10.1186/s12903-020-01227-y (PMC7469363; doi:10.1186/s12903-020-01227-y)
Supplement: Supplementary file 1 — Additional file 1. Study questionnaire, semi-structured questionnaire prepared in order to extract the following data: socio-demographic, clinical characteristics, treatment type and dose, and treatment satisfaction. [file 12903_2020_1227_MOESM1_ESM.docx]

**Protocol title**

- Clinical Characteristics and Associated factors of Trigeminal Neuralgia in Addis Ababa, Ethiopia: Experience from Addis Ababa, Ethiopia

| **PART I: Socio Demographic Data** | |
| --- | --- |
| 1. I-CARE Number | |
| 1. Gender A. Male B. Female | |
| 1. Age | |
| 1. Duration of illness | |
| 1. Classification of TN A. Classical TN B. Secondary TN | |
| **PART II: Trigeminal neuropathy related clinical characteristics** | |
| 1. Which branch of Trigeminal Nerve is involved? | 1) Ophthalmic (V1)  2) Maxillary (V2)  3) Mandibular (V3)  4) V2 + V3  5) V1 + V2 + V3 |
| 1. Cranial nerve examination finding | Neurological examination  a) Normal  b) Abnormal, specify______________________  C) Other neurologic examination findings___________________ |
| 1. Quality of pain   (patients could have more than one) | 1) Feeling of being injected with red hot needle  2) Burning  3) Sharpe and shooting 4) pulling type  5) Electric shock like  6) Mixed quality  6) Others……………… |
| 1. Is there any trigger zone? | 1)Yes, where? ______________________________  2. No |
| 1. Is there a triggering factor?   (Patients could have more than one) | 1) Talking 6) Washing  2) Swallowing 7) Shaving  3) Touching 8) Laughing  4) Cold wind blowing 9) Chewing  5) Mouth opening 10) Drinking hot or cold drinks  11) Mixed factors |
| 1. Is there any autonomic phenomenon during attack? | [0] No  [1] Yes, which one among the following?  1) Lacrimation 2) Conjunctival hyperemia  3) Rhinorrhea 4) Ptosis |
| 1. Did you ever experience attack during sleep? | 1. Yes 2. No |
| 1. Does the pain worsen during particular seasons? | 1. No 2. Yes. Specify_____________________ |
| 1. List of medications patients is taking (including dose) | 1)_________________________  2)__________________________  3)____________________________ |
| 1. Treatment satisfaction | 1) No satisfaction  2) Mild  3) Prominent satisfaction (moderate + good) |
| 1. Past dental extraction history for the pain management? | 1.No  2.Yes, specify __________________ |
| 1. Brain MRI or CT scan | 1. No 2. Yes, if yes specify_______________________________ |
| 1. Family history of Trigeminal neuralgia? | 1. Yes 2. No |
